# Plasma Levels of High Sensitivity Cardiac Troponin T in Adults with Repaired Tetralogy of Fallot

**DOI:** 10.1038/srep14050

**Published:** 2015-09-11

**Authors:** Clare T. M. Lai, Sophia J. Wong, Janice J. K. Ip, Wai-keung Wong, Kwong-cheong Tsang, Wendy W. M. Lam, Yiu-fai Cheung

**Affiliations:** 1 Division of Paediatric Cardiology, Department of Paediatrics and Adolescent Medicine, Queen Mary Hospital, The University of Hong Kong, Hong Kong, China; 2 Department of Radiology, Queen Mary Hospital, Hong Kong, China; 3 Department of Pathology and Clinical Biochemistry, Queen Mary Hospital, Hong Kong, China

## Abstract

Detectable low circulating level of cardiac troponin T (cTnT) may reflect subclinical myocardial injury. We tested the hypothesis that circulating levels of hs-cTnT are altered in adults with repaired tetralogy of Fallot (TOF) and associated with ventricular volume load and function. Eighty-eight TOF patients and 48 controls were studied. Plasma hs-cTnT levels were determined using a highly sensitive assay (hs-cTnT). The right (RV) and left ventricular (LV) volumes and ejection fraction (EF) were measured using 3D echocardiography and, in 52 patients, cardiac magnetic resonance (CMR). The median (interquartile range) for male and female patients were 4.87 (3.83–6.62) ng/L and 3.11 (1.00–3.87) ng/L, respectively. Thirty percent of female but none of the male patients had increased hs-cTnT levels. Female patients with elevated hs-cTnT levels, compared to those without, had greater RV end-diastolic and end-systolic volumes and LV systolic dyssynchrony index (all p < 0.05). For patient cohort only, hs-cTnT levels correlated positively with CMR-derived RV end-diastolic volume and negatively with echocardiography-derived LV and RV EF (all p < 0.05). Multiple linear regression identified sex and RV EF as significant correlates of log-transformed hs-cTnT levels. Increased hs-cTnT levels occur in 30% of female patients after TOF repair, and are associated with greater RV volumes and worse RV EF.

Dysfunction of the right and left ventricles remains to be an issue of concern in adults with repaired tetralogy of Fallot (TOF)^1^,^2^,^3^. In these patients, several factors may contribute to myocardial damage and cause progressive ventricular remodeling and dysfunction. These include altered expression of genes associated with apoptosis, remodeling, and myocardial contractility^4^, failure of hypoxic adaptation of the right ventricle^5^, chronic pulmonary regurgitation^1^,^6^, increased right ventricular (RV) afterload due to pulmonary arterial stenosis, ventricular fibrosis^7^, and adverse ventricular-ventricular interaction^2^,^8^,^9^.

Recently, quantification of minute amount of cardiac troponin T, a circulating marker of myocardial injury, has been made possible using a highly sensitive assay (hs-cTnT)^10^. In a general population without overt cardiovascular disease, the level of hs-cTnT has been shown to be related to cardiovascular risk factors, structural alteration of the left ventricle, and risk for all-cause mortality^11^,^12^. In patients with heart failure due to different aetiologies including congenital heart disease, the rate of adverse cardiac events has been reported to be significantly greater in patients with high circulating levels of cTnT^13^. Detectable circulating cTnT, albeit at a low level, has been implicated to reflect subclinical myocardial injury^14^,^15^.

The clinical relevance of hs-cTnT in the setting of RV volume overload and dysfunction in patients with repaired TOF has, however, not been explored. Nonetheless, increased level of plasma hs-cTnT found in adults with pulmonary arterial hypertension secondary to congenital heart disease^16^ suggests its potential reflection of RV myocardial injury. In this study, we tested the hypothesis that circulating levels of hs-cTnT are altered in adults with repaired TOF and associated with ventricular volume load and function.

## Methods

### Subjects

This is a prospectively study. Eighty-eight adults with repaired TOF were recruited consecutively from the congenital heart clinic. All of the invited patients agreed to participate in the study. Clinical data including age at and type of surgical repair, duration of follow-up since surgery, current cardiac medications, renal dysfunction, documented systemic hypertension, and smoking status were obtained from case records. Forty-eight healthy adults were recruited as controls. These are healthy siblings of patients and volunteering adult hospital staff members and their friends. The body weight and height were measured and the body surface area was calculated accordingly. All experimental protocols were approved by the Institutional Review Board of The University of Hong Kong/Hospital Authority West Cluster, Hong Kong, and all of the subjects gave informed written consent. This study and the methods as described were performed in accordance with the ethical standards laid down in the 1964 Declaration of Helsinki and its later amendments.

### hs-cTnT assay

Venous blood was collected from all of the subjects on the day of echocardiographic assessment. Plasma samples were stored at −80 °C until assay. Plasma level of hs-cTnT was measured using the Elecsys-2010 Troponin T hs platform (Roche Diagnostics), which has a lower detection limit of 3 ng/L.

### Echocardiographic assessment

Transthoracic echocardiographic assessment was performed using the Vivid 7 ultrasound system (General Electric, Horten, Norway). Measurements of echocardiographic parameters were made over three cardiac cycles and the average was used for statistical analysis. Severity of tricuspid regurgitation^17^ and pulmonary regurgitation^18^ was graded semi-quantitatively by colour Doppler imaging. Real-time 3-dimensional echocardiographic measurement was performed using the matrix array transducer (Vivid 7 ultrasound system, General Electric) as previously described^19^. Offline analysis was performed using commercial 4-dimensional analysis software (Tomtec Imaging Systems, Unterschleisheim, Germany). The RV and LV volumes were measured, indexed by body surface area, and ejection fractions of the respective ventricles were calculated accordingly. Left ventricular mechanical dyssynchrony was quantified by calculation of the systolic dyssynchrony index (SDI)^20^. We have reported previously on low intra- and inter-observer variability in the measurements of RV and LV ejection fraction and LV SDI^19^.

### Cardiac magnetic resonance

Fifty-two (23 males, 29 females) patients agreed to undergo further cardiac magnetic resonance (CMR) for determination of pulmonary regurgitant fraction, ventricular end-systolic and end-diastolic volumes and ejection fraction. The interval between CMR examination and echocardiographic and blood investigations was within 6 months. The examination was performed using a 1.5-T superconducting whole-body imager (GE Signa Horizon Echospeed, General Electric Medical Systems, Milwaukee, Wisconsin, USA) with a phase-array torso coil. ECG-trigger fast spin-echo double inversion recovery axial procedure (TR/TE, auto/42) was performed for baseline imaging. Analysis of ventricular function was performed by fastcard-SPGR cine on axial and short-axis planes. Flow analysis of the pulmonary artery was obtained by using fast 2D phase contrast and taking a plane perpendicular to the flow direction of the RV outflow tract just above the pulmonary valve before bifurcation. Analyses of ventricular function and flow were performed using the software in Advantage Window version 4.2.

### Statistical analysis

Normally distributed data are presented as mean ± SD. Plasma levels of hs-cTnT had a non-parametric distribution and are presented as median (interquartile range). Demographic and echocardiographic parameters of patients and controls were compared using unpaired Student’s t test and Fisher’s exact test where appropriate. An undetectable level of hs-cTnT was assigned a value of 1 ng/L, which gives a value of 0 after logarithmic transformation. A similar approach to assign a level of 1.5 ng/L for undetectable levels for continuous model analysis has also been previously^21^,^22^. Comparison of plasma hs-cTnT levels between patients and controls was performed using Mann-Whitney U test. The upper limits of normal hs-cTnT for each of the genders were derived from non-parametric estimation of the 99th percentile based on female and male controls. The female and male patients were stratified into two groups based on the respective cutoff values: group I with increased hs-cTnT levels >99^th^ centile and group II with hs-cTnT levels ≤99^th^ centile. Differences in variables between the two subgroups were compared using Student’s *t* test and Fisher’s exact test where appropriate. Correlations between log-transformed hs-cTnT level and ventricular volumes and ejection fraction were performed using Pearson correlation. Multiple linear regression analysis was used to identify significant determinants of log-transformed hs-cTnT levels in patients. Statistical analyses were performed with SPSS, version 16.0 (SPSS Inc., Chicago IL, USA.). A p value < 0.05 was considered statistically significant.

## Results

### Subjects

The 88 (44 females) patients, aged 24.0 ± 6.4 years, were studied at 19.1 ± 6.4 years after TOF repair. Twelve patients required initial palliation by a systemic-pulmonary arterial shunt. For the total surgical repair, 76 patients had transannular patch repair of RV outflow, 3 had insertion of a non-valved conduit, and 1 had a valved-conduit implanted. Fourteen patients had branch pulmonary arterial stenosis on follow-up, which required balloon angioplasty in 14 and stent implantation eventually in 3. Two patients had undergone radiofrequency ablation for atrial flutter. None of the patients had coronary anomalies and none required pacemaker implantation. All of the patients were in sinus rhythm at the time of study. Four patients, all females, were on cardiac medications, which included digoxin (n = 3), diuretics (n = 2), warfarin (n = 1), beta-blocker (n = 1), and amiodarone (n = 1). Two male patients were smokers. None of our patients had documented renal dysfunction or systemic hypertension. The 48 controls (22 females) were aged 22.8 ± 4.7 years (p = 0.27). Body weight (53.2 ± 9.8 kg vs 60.7 ± 13.6 kg, p = 0.001) and body height (1.6 ± 9.2 m vs 1.7 ± 10.2 m, p < 0.001) were significantly lower in patients than controls.

### Plasma hs-cTnT levels

Cardiac troponin T was detectable by the highly sensitive assay in 63 (71.6%) patients and in 24 (49.0%) controls (p = 0.01). The median plasma level of hs-cTnT was significantly higher in patients (3.87 ng/L, interquartile range 1.00–5.21 ng/L) than controls (1.00 ng/L, interquartile range 1.00–4.25 ng/L, p = 0.01).

For males, the prevalence of detectable plasma hs-cTnT was similar between patients and controls (90.9% vs 84.6%, p = 0.46). However, for females, the prevalence of detectable plasma hs-cTnT was significantly greater in patients than controls (52.3% vs 9.1%, p = 0.001).

**Figure 1:**
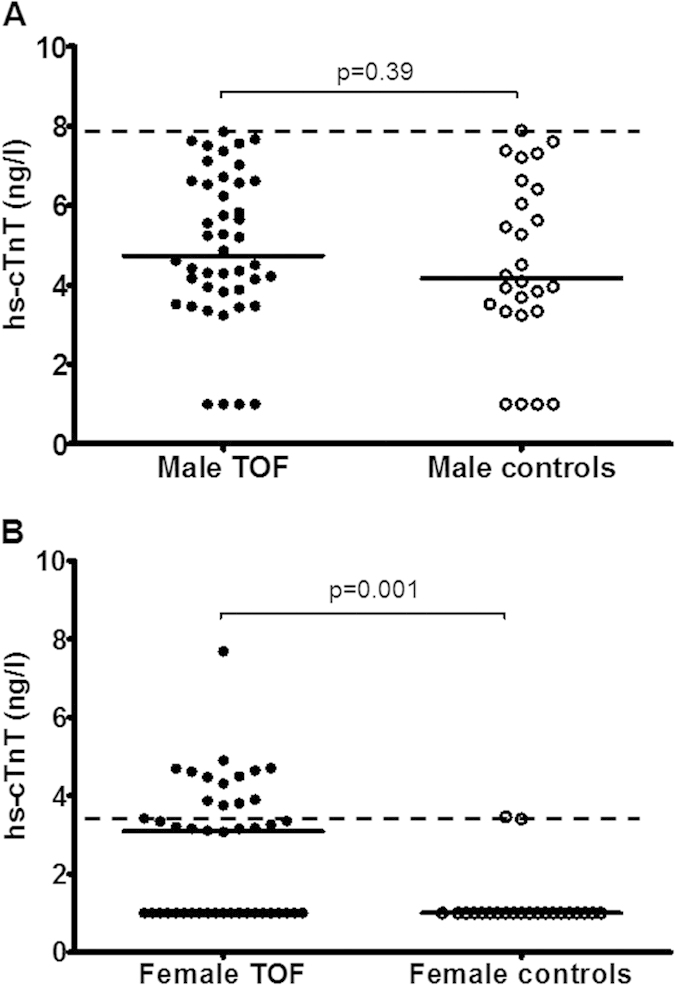
Scatter plots showing plasma high sensitivity cardiac troponin T (hs-cTnT) levels in (A) male and (B) female patients after repair of tetralogy of Fallot (TOF) and control subjects. Solid lines represent the median, dotted lines represent the 99^th^ percentile derived from non-parametric estimation.

The distribution of plasma levels of hs-cTnT differed between males and females (Fig. 1). In males, the median plasma hs-cTnT level was similar between patients (4.87 ng/L, interquartile range 3.83 to 6.62 ng/L) and controls (4.25 ng/L, interquartile range 3.83 to 6.62 ng/L, p = 0.39). For the two male smokers, their plasma hs-cTnT levels (4.31 and 5,75 ng/L) remained with the interquartile range for controls. Female patients, however, had significantly higher median plasma hs-cTnT level (3.11 ng/L, interquartile range 1.00 to 3.87 ng/L) than controls (1.00 ng/L, interquartile range 1.00 to 1.00 ng/L).

### Echocardiographic findings

Tricuspid regurgitation was mild in 47 and trace in 41 patients, while pulmonary regurgitation was moderate to severe in 35, mild in 37, and trace to absent in 16. Table 1 shows the demographic variables and echocardiographic findings in patients and controls stratified by sex gender. Patients of both genders had significantly greater RV end-systolic volume, end-diastolic volume, and ejection fraction, and LV end-systolic volume and SDI compared with gender-specific controls (all p < 0.05). Among patients who had undergone CMR, male patients were found to have greater LV end-systolic (p = 0.03) and end-diastolic (p = 0.04) volumes compared with female patients.

### Plasma hs-cTnT levels and ventricular size and function

Of the 44 female patients, 13 (30%) had a plasma hs-cTnT level above the 99^th^ percentile cutoff value of 3.46 ng/L. Comparisons of demographic, clinical, and cardiac functional parameters between female patients with and those without significantly increase in plasma hs-cTnT levels are shown in Table 2. Although the two groups had similar demographic and clinical parameters, females with higher plasma hs-cTnT levels had significantly greater RV end-diastolic and end-systolic volumes based on both 3-dimensional echocardiography (p = 0.035 and 0.018, respectively) and CMR (p = 0.027 and 0.008, respectively) and LV mechanical dyssynchrony (p = 0.018).

**Figure 2:**
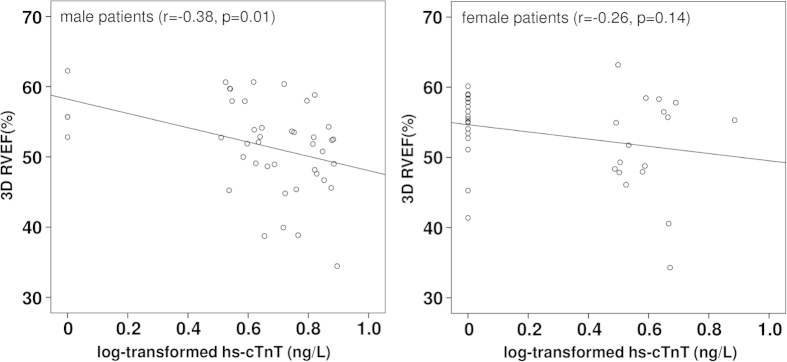
Scatter plots showing a negative correlation between log-transformed hs-cTnT levels and echocardiographically-derived right ventricular (RV) ejection fraction (EF) in male and female patients.

For the whole patient cohort, plasma hs-cTnT level correlated negatively with echocardiographically-derived RV (r = −0.33, p = 0.004) and LV (r = −0.24, p = 0.032) ejection fraction but not with the severity of tricuspid (p = 0.80) or pulmonary regurgitation (p = 0.83). By gender, plasma hs-cTnT level showed negative correlations with RV ejection fraction in both male (r = −0.38, p = 0.012), and female (r = −0.26, p = 0.14) patients (Fig. 2). There were no associations between plasma hs-TnT level and RV ejection fraction in either male or female controls (supplementary Fig. 1). For patients who had undergone CMR, plasma hs-cTnT level was found to correlate positively with RV end-diastolic volume (r = 0.33, p = 0.02).

Based on the entire cohort of 88 patients and with the use of echocardiographically-derived ventricular volumes and ejection fraction, multiple stepwise linear regression was performed to identify significant correlates of log-transformed hs-cTnT levels. The significant independent correlates were sex gender (*β* = 0.57, p < 0.001) and RV ejection fraction (*β* = −0.29, p = 0.002) after adjustment for age at study, duration of follow-up, RV and LV end-systolic and diastolic volumes, and LV SDI.

## Discussion

The present study demonstrates increased plasma level of hs-cTnT and its association with greater RV volume load, lower RV ejection fraction, and greater LV mechanical dyssynchrony in adult female patients with repaired TOF. Even after adjustment of sex gender by multivariate analysis, hs-cTnT level remains to be an independent correlate of RV ejection fraction. To our knowledge, this is the first study to determine plasma levels of hs-cTnT in patients late after TOF repair.

Differences in circulating levels of hs-cTnT between males and females are well documented^10^,^11^,^23^. Meaningful interpretation of cTnT levels in our patients hence necessitates gender-specific comparisons. The male cutoff value of 7.86 ng/L, based on non-parametric estimation of the 99^th^ percentile in the present study, corresponds to the highest tertile of hs-cTnT levels in a recent Japanese study^12^. In this Japanese study, the cut-off tertile values for middle-aged men without overt cardiovascular disease were respectively ≤2 ng/L, 3 to 4 ng/L, and ≥5 ng/L. Our finding of a high prevalence of detectable hs-cTnT in about 85% of males is also comparable to the 81% reported in the previous study. Data on the distribution of hs-cTnT level are available in healthy middle-age^24^,^25^ but not young adult females. In a Caucasian population study, the 13% prevalence of detectable plasma hs-cTnT in women younger than 40 years^11^ is comparable to the finding of about 9% in our female controls. Although reference range for the hs-cTnT assay in young adults remains to be established, minimal elevation of cTnT levels to 3 to 5 ng/L has been associated with an increase in all-cause mortality in the general population without known coronary heart disease or stroke^21^.

Despite adjustment of sex gender by multivariate analysis of the entire studied cohort, hs-cTnT level remains to be an independent correlate of RV ejection fraction. Furthermore, among female patients, increased hs-cTnT levels are associated with greater RV volume load, lower RV ejection fraction, and greater LV mechanical dyssynchrony. In patients with heart failure, hs-cTnT has been reported to correlate with LV ejection fraction and parameters of RV dysfunction^26^. In the general population, de Lemos *et al.* further demonstrated a negative association between circulating hs-cTnT levels and LV ejection fraction^11^. With regard to right-sided pathologies, increased hs-TnT levels have been shown to correlate with RV systolic dysfunction in patients with pulmonary arterial hypertension^27^ and acute pulmonary embolism^28^.

The findings of a greater prevalence of detectable plasma hs-cTnT and significantly higher plasma levels in female patients with repaired TOF compared with gender-specific controls are intriguing. Gender differences in the outcomes of congenital heart disease have been reported^29^,^30^,^31^. Higher odds of death in female compared with male children undergoing cardiac surgery have been shown, although the cause is unknown^32^. In the CONgenital CORvitia (CONCOR) Dutch national registry reporting on long-term outcomes of adult congenital heart patients, women were found to have a 33% higher risk of pulmonary hypertension^31^. This has been thought to be related to the smaller arteries and more frequent development of endothelial and smooth cell dysfunction^33^. Whether these factors may contribute to increased RV volume load and subclinical myocardial injury in female TOF patients requires further research. Interestingly, the female gender has been found to be a risk factor for early RV insufficiency as defined by age-matched gender-specific standard deviation score of RV ejection fraction after TOF repair^34^. Taken together, it is tempting to speculate that female patients might be more vulnerable to subclinical myocardial injury in the setting of chronic RV volume overload post TOF repair.

The mechanisms underlying the increase in hs-cTnT level in this subgroup of patients remain unclear. Reduced RV myocardial microvascular density^35^ and myocardial perfusion reserve^36^ reported in repaired TOF patients may predispose to myocardial ischaemia during stress. In isolated rat hearts, increased preload has been shown to induce degradation of troponin I^37^. Furthermore, myocardial stretch-induced calpain activation and troponin degradation, with release of troponin fragments, have been hypothesized as plausible mechanisms of progressive deterioration of cardiac function in heart failure^38^. Albeit speculative, chronic stretching of the RV myocardium in repaired TOF patients with superimposed vulnerable factors reported in females as alluded to earlier may be a possible explanation.

Cardiovascular risk factors including hypertension, renal dysfunction, and smoking have been shown to be independent determinants of hs-cTnT levels^12^. Systemic hypertension is not typically observed in patients with repaired TOF in the long term. Review of clinical records did not reveal the presence of these potential confounding risk factors, with the exception of smoking being reported in two male patients. Future longitudinal studies to evaluate the prognostic significance of increased hs-TnT after TOF repair should include prospective collection of these data.

Several limitations warrant further comments. Firstly, it is not the intention of this study to define a cut-off level of hs-cTnT for diagnosing subpulmonary RV dysfunction. Rather, our study serves to provide evidence of subclinical myocardial injury that may represent a new perspective in the understanding of progression of RV dysfunction late after TOF repair. Secondly, the case-control design does not permit evaluation of the prognostic value of increased hs-cTnT levels in patients. The number of controls studied was smaller than that of patients. This reflects perhaps the challenge of recruiting healthy subjects into studies that requiring blood taking. Thirdly, we have examined a rather limited clinical spectrum of adult TOF patients who are young and have relatively preserved ventricular function. The true impact of aging and pregnancy on hs-cTnT levels and RV size and function has also not been explored.

In conclusion, this study provides the first evidence that plasma hs-cTnT levels are increased in a subgroup of adult females late after repair TOF, and are associated with greater RV volume overload and worse RV systolic function. Further studies are warranted to define whether female TOF patients are more vulnerable to functional compromise of the right ventricle.

## Additional Information

**How to cite this article**: Lai, C. T. M. *et al.* Plasma Levels of High Sensitivity Cardiac Troponin T in Adults with Repaired Tetralogy of Fallot. *Sci. Rep.* **5**, 14050; doi: 10.1038/srep14050 (2015).

## Supplementary Material

Supplementary Information

## Figures and Tables

**Table 1:** Demographic, clinical, and cardiac parameters in patients and controls by gender.

	Male patients (I) (n = 44)	Female patients (II) (n = 44)	Male controls (III) (n = 26)	Female controls (IV) (n = 22)	p_1_ (I vs II)	p_2_ (I vs III)	p_3_ (II vs IV)
Demographic parameters							
Age (years)	23.6 ± 6.6	24.3 ± 6.3	23.3 ± 5.5	22.0 ± 3.7	0.63	0.84	0.12
Weight (kg)	56.1 ± 9.0	50.3 ± 9.9	69.0 ± 12.6	51.0 ± 6.2	0.005*	<0.001*	0.79
Height (m)	1.7 ± 7.7	1.6 ± 7.0	1.8 ± 7.3	1.6 ± 6.5	<0.001*	<0.001*	0.017*
Clinical parameters							
Need for medications	0/44	4/44			0.009*		
Age at surgery (years)	4.4 ± 4.5	4.4 ± 3.6			0.99		
Duration after repair (years)	19.3 ± 6.8	19.0 ± 6.0			0.82		
Echocardiographic parameters							
RV EDV (mL/m^2^)	121 ± 45	118 ± 44	71 ± 15	59 ± 15	0.33	<0.001*	<0.001*
RV ESV (mL/m^2^)	61 ± 28	63 ± 22	30 ± 7	24 ± 6	0.41	<0.001*	<0.001*
RV EF (%)	51 ± 7	53 ± 6	57 ± 4	58 ± 4	0.20	<0.001*	0.007*
LV EDV (mL/m^2^)	67 ± 16	63 ± 23	64 ± 10	55 ± 13	0.34	0.35	0.10
LV ESV (mL/m^2^)	32 ± 8	31 ± 12	28 ± 5	25 ± 7	0.69	0.01*	0.022*
LV EF (%)	52 ± 8	55 ± 7	57 ± 4	55 ± 4	0.15	0.001*	0.63
LV SDI (%)	7.5 ± 3.8	7.4 ± 3.4	4.0 ± 0.9	3.9 ± 0.8	0.91	<0.001*	<0.001*
Cardiac magnetic resonance	(n = 21/44)	(n = 29/44)					
RV EDV (mL/m^2^)	156 ± 52	140 ± 38			0.22		
RV ESV (mL/m^2^)	83.5 ± 33.4	74.0 ± 30.7			0.31		
RV EF (%)	49.4 ± 8.4	50.1 ± 8.0			0.75		
PRF (%)	32.4 ± 17.8 (n = 23/44)	37.5 ± 15.1 (n = 28/44)			0.65		
LV EDV (mL/m^2^)	76.1 ± 18.0	66.0 ± 14.0			0.03*		
LV ESV (mL/m^2^)	32.4 ± 8.5	26.9 ± 9.8			0.04*		
LV EF (%)	56.8 ± 9.7	60.3 ± 7.4			0.15		

Abbreviations: EDV, end-diastolic volume; EF, ejection fraction; ESV, end-systolic volume; LV, left ventricular; PRF, pulmonary regurgitant fraction; RV, right ventricular; SDI, systolic dyssynchrony index. *Statistically significant.

**Table 2:** Comparisons of demographic, clinical, and cardiac parameters between female patients with plasma hs-cTnT levels above versus those with levels below the 99^th^ percentile cutoff.

	≤3.46 ng/L (n = 31)	>3.46 ng/L (n = 13)	p
Demographic parameters			
Age (years)	24.4 ± 6.0	24.0 ± 7.3	0.83
Weight (kg)	51.4 ± 9.3	47.8 ± 11.2	0.27
Height (m)	1.6 ± 7.4	1.5 ± 6.0	0.49
Clinical parameters			
Need for medications	3	1	1.00
Age at surgery (years)	4.75 ± 4.20	3.58 ± 1.29	0.37
Duration after repair (years)	19.7 ± 6.1	17.1 ± 5.8	0.22
Echocardiographic parameters			
RV EDV (mL/m^2^)	109 ± 37	143 ± 51	0.035*
RV ESV (mL/m^2^)	50 ± 18	71 ± 32	0.018*
RV EF (%)	54 ± 5	51 ± 8	0.29
LV EDV (mL/m^2^)	62 ± 24	68 ± 18	0.49
LV ESV (mL/m^2^)	30 ± 13	33 ± 11	0.55
LV EF (%)	55 ± 6	52 ± 6	0.16
LV SDI (%)	6.7 ± 2.8	9.6 ± 4.1	0.018*
Cardiac magnetic resonance			
(n = 29/44)			
RV EDV (mL/m^2^)	130 ± 28	163 ± 48	0.027*
RV ESV (mL/m^2^)	64 ± 19	96 ± 41	0.008*
RV EF (%)	52 ± 5.3	46 ± 11	0.14
PRF (%)	37 ± 18	38 ± 8	0.96
(n = 28/44)			
LV EDV (mL/m^2^)	66 ± 13	66 ± 16	0.95
LV ESV (mL/m^2^)	26 ± 9	28 ± 12	0.74
LV EF (%)	61 ± 6	59 ± 9	0.65

Abbreviations as in Table 1. *Statistically significant.
