# Supplementary material for: Plasma Levels of High Sensitivity Cardiac Troponin T in Adults with Repaired Tetralogy of Fallot
Source: Sci Rep. 2015 Sep 11;5:14050. doi: 10.1038/srep14050 (PMC4566090; doi:10.1038/srep14050)
Supplement: Supplementary Information [file srep14050-s1.pdf]

Plasma Levels of High Sensitivity Cardiac Troponin T in Adults with Repaired Tetralogy of Fallot

Clare TM Lai[1], Sophia J Wong[1], Janice JK Ip[2], Wai-keung Wong[3], Kwong-cheong Tsang[3], Wendy WM Lam[2], and Yiu-fai Cheung[1,*]

[1]Division of Paediatric Cardiology, Department of Paediatrics and Adolescent Medicine, Queen Mary Hospital, The University of Hong Kong, [2]Department of Radiology, Queen Mary Hospital, and [3]Department of Pathology and Clinical Biochemistry, Queen Mary Hospital, Hong Kong, China

Supplementary Figure 1

Scatter plots showing the lack of correlations between log-transformed hs-cTnT levels and echocardiographically-derived right ventricular (RV) ejection fraction (EF) in male and female controls.

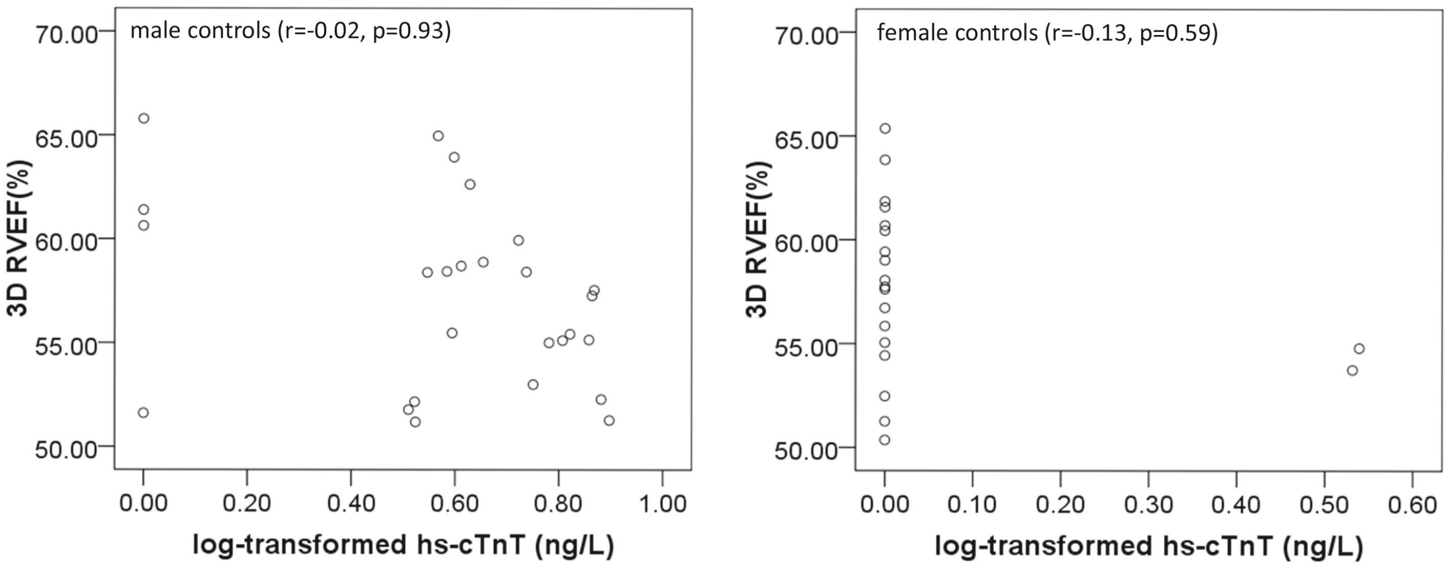
male controls (r=-0.02, p=0.93)
3D RVEF(%)
70.00
65.00
60.00
55.00
50.00
0.00
0.20
0.40
0.60
0.80
1.00
log-transformed hs-cTnT (ng/L)
female controls (r=-0.13, p=0.59)
3D RVEF(%)
70.00
65.00
60.00
55.00
50.00
0.00
0.10
0.20
0.30
0.40
0.50
0.60
log-transformed hs-cTnT (ng/L)
